# Supplementary material for: New Species of Didymellaceae within Aquatic Plants from Southwestern China
Source: J Fungi (Basel). 2023 Jul 19;9(7):761. doi: 10.3390/jof9070761 (PMC10381294; doi:10.3390/jof9070761)
Supplement: Supplementary file 1 [file jof-09-00761-s001.zip › Supplementary Table 1.pdf]

Table S1. GenBank accession numbers of taxa used in phylogenetic analyses

Species	Strain number	GenBank accession numbers			
		ITS	LSU	*rpb2*	*tub2*
Allophoma alba	CBS 120422	MN973469	MN943671	MT018044	MT005568
Allophoma anatii	CBS 124673	MN973472	MN943674	MT018048	MT005571
Allophoma cylindrispora	CBS 142453	LT592920	LN907376	LT593058	LT592989
Allophoma hayatii	YMF1.05027	MH257423	MH257424	OQ737920	MH400428
Allophoma hayatii	CBS 142859	KY684812	KY684814	MF095108	KY684816
Allophoma hayatii	CBS 142860	KY684813	KY684815	MF095109	KY684817
Allophoma labilis	CBS 479.93	GU237868	GU238092	MN983277	GU237620
Allophoma labilis	CBS 540.77	MN972686	-	MN983278	MN983703
Allophoma minor	CBS 325.82	GU237831	GU238107	KT389553	GU237632
Allophoma nicaraguensis	CBS 506.91	GU237876	GU238058	KT389551	GU237596
Allophoma oligotrophica	CGMCC 3.18114	KY742040	KY742194	KY742128	KY742282
Allophoma piperis	CBS 268.93	GU237816	GU238129	KT389554	GU237644
Allophoma siamensis	MFLUCC 17-2422	MK347742	MK347959	MK434912	MK412867
Allophoma tropica	CBS 436.75	GU237864	GU238149	KT389556	GU237663
Allophoma zantedeschiae	CBS 229.32	KT389473	KT389690	KT389558	KT389767
Ascochyta fabae	CBS 524.77	GU237880	GU237963	MT018241	GU237526
Ascochyta lentis	CBS 370.84	KT389474	KT389691	MT018246	KT389768
Ascochyta pisi	CBS 122785	GU237763	GU237969	MT018244	GU237532
Ascochyta syringae	CBS 545.72	KT389483	KT389700	MT018245	KT389777
Ascochyta viciae	CBS 451.68	KT389484	KT389701	KT389562	KT389778
Ascochyta viciae-pannonicae	CBS 254.92	KT389485	KT389702	MT018250	KT389779
Ascochyta viciae-villosae	CBS 255.92	MN973584	MN943790	MT018249	MT005690
Boeremia coffeae	CBS 109183 (R)	GU237748	GU237943	KT389566	GU237505

Boeremia crinicola	CBS 109.79 (R)	GU237737	GU237927	KT389563	GU237489
Boeremia diversispora	CBS 102.80 (R)	GU237725	GU237930	KT389565	GU237492
Boeremia exigua	CBS 107.21	KT389491	KT389708	-	KT389785
Boeremia exigua	CBS 119.38	KT389490	KT389707	KT389583	KT389784
Boeremia exigua	CBS 101197	MN972715	GU237931	MN983312	MN983731
Boeremia exigua var. *exigua*	CBS 101150	GU237715	EU754182	KT389568	GU237495
Boeremia exigua var. *forsythiae*	CBS 101213	GU237723	GU237932	KT389571	GU237494
Boeremia exigua var. *gilvescens*	CBS 761.70	MH859929	MH871730	-	MN983778
Boeremia exigua var. *gilvescens*	PD 98/213	KY484653	-	-	KY484752
Boeremia exigua* var. *kasensis	**YMF1.05091**	**MH233939**	**MH257435**	**MH311790**	**MH422975**
Boeremia exigua* var. *kasensis	**YMF1.05031**	**MH233941**	**MH257430**	**MH311787**	**MH400434**
Boeremia exigua* var. *kasensis	**YMF1.05057**	**MH233944**	**MH257426**	**MH311784**	**MH400430**
Boeremia exigua* var. *kasensis	**YMF1.05205**	**MH233946**	**MH257439**	**MH311793**	**MH422979**
Boeremia exigua* var. *kasensis	**YMF1.05043**	**MH233947**	**MH257440**	**MH311794**	**MH422980**
Boeremia exigua* var. *kasensis	**YMF1.05094**	**MH233943**	**MH257437**	**MH311791**	**MH422977**
Boeremia exigua* var. *kasensis	**KS18**	**MH233945**	**MH257428**	**-**	**MH400432**
Boeremia exigua var. *populi*	CBS 113.36	MN972774	MH867235	MN983373	MN983790
Boeremia exigua var. *populi*	CBS 120111	MN972773	-	MN983372	MN983789
Boeremia exigua var. *pseudolilacis*	CBS 118803	MN972787	-	MN983388	MN983804
Boeremia exigua var. *pseudolilacis*	CBS 140180	MN972786	-	MN983387	MN983803
Boeremia exigua var. *viburni*	YMF1.05077	MH233934	MH257425	MH311783	MH400429
Boeremia exigua var. *viburni*	YMF1.05216	MH233958	MH257448	MH311800	MH422988
Boeremia exigua var. *viburni*	YMF1.05009	MH233957	MH257449	MH311801	MH422989
Boeremia exigua* var. *vulgartis	**YMF1.05039**	**MH233959**	**MH257450**	**MH311802**	**MH422990**
Boeremia exigua* var. *vulgartis	**YMF1.05042**	**MH233960**	**MH257451**	**MH311803**	**MH422991**
Boeremia exigua* var. *vulgartis	**YMF1.05206**	**MH233951**	**MH257442**	**-**	**MH422982**

Boeremia exigua* var. *vulgartis	**YL23**	**MH233961**	**MH257452**	**MH311804**	**MH422992**
Boeremia exigua var. *vibumi*	CBS 100354	GU237711	GU237944	KT389577	GU237506
Boeremia exigua var. *viburni*	CBS 101211	GU237722	GU237945	MN983334	GU237507
Boeremia foveata	CBS 200.37*	MW810263	MW715034	MW735651	MW815124
Boeremia galiicola	MFLUCC15-0771	KX698037	KX698026	-	KX698030
Boeremia hedericola	CBS 367.91	GU237842	GU237949	KT389579	GU237511
Boeremia heteromorpha	CBS 443.94*	GU237866	GU237935	KT389573	GU237497
Boeremia lilacis	CBS 569.79 (R)	GU237892	GU237936	-	GU237498
Boeremia linicola	YMF1.05017	MH233950	MH257441	MH311795	MH422981
Boeremia linicola	CBS 116.76 (R)	GU237754	GU237938	KT389574	GU237500
Boeremia linicola	CBS 114.28	MH854944	MH866435	MN983365	GU237499
Boeremia lycopersici	CBS 161.47*	MN972797	NG 077375	MW735649	MN983814
Boeremia noackiana	CBS 100353 (R)	GU237710	GU237952	-	GU237514
Boeremia opuli	CGMCC38354	KY742045	KY742199	KY742133	KY742287
Boeremia opuli	LC8118	KY742046	KY742200	KY742134	KY742288
Boeremia populi	CBS 100167*	GU237707	GU237939	-	GU237501
Boeremia pseudolilacis	CBS 423.67	KT389487	KT389704	KT389576	KT389781
Boeremia pseudolilacis	CBS 462.67	KT389488	KT389705	MN983420	KT389782
Boeremia sambuci-nigrae	CBS 629.68*	GU237897	GU237955	-	GU237517
Boeremia strasseri	CBS 126.93 (R)	GU237773	GU237956	KT389584	GU237518
Boeremia telephii	CBS 109175 (R)	GU237741	GU237958	KT389585	GU237520
Boeremia trachelospermi	CGMCC 38222	KY064028	KY064032	KY064033	KY064051
Briansuttonomyces eucalypti	CBS 114879	KU728479	KU728519	MT018239	KU728595
Briansuttonomyces eucalypti	CBS 114887	KU728480	KU728520	MT018240	KU728596
Calophoma aquilegiicola	CBS 107.96	GU237735	GU238041	KT389586	GU237581
Calophoma clematidis-rectae	CBS 507.63	FJ515606	FJ515647	KT389589	FJ515624

Calophoma glaucii	CBS 114.96	FJ515609	FJ515649	MT018232	FJ515627
Calophoma rosae	CGMCC 3.18347	KY742049	KY742203	KY742135	KY742291
Cumuliphoma indica	CBS 991.95	MN973546	MN943753	MT018184	MT005649
Cumuliphoma indica	CBS 123396	FJ427046	MN973198	MN983433	MN983839
Cumuliphoma lijiangensis	**YMF1.05096**	**MH257400**	**MH257485**	**MH311838**	**MH423002**
Cumuliphoma omnivirens	CBS 341.86	FJ427042	LT623214	LT623260	FJ427152
Cumuliphoma pneumoniae	CBS 142454	LT592925	LN907392	LT593063	LT592994
Didymella acetosellae	CBS 631.76 ET	MN973542	MN943749	MT018176	MT005645
Didymella aloeicola	CBS 562.88 T	MN973535	MN943742	MT018164	MT005638
Didymella americana	CBS 185.85	FJ426972	GU237990	KT389594	FJ427088
Didymella americana	CBS 568.97	FJ426974	GU237991	MN983437	FJ427090
Didymella anserina	CBS 360.84	GU237839	GU237993	KT389596	GU237551
Didymella aquatica	CGMCC 3.18349 T	KY742055	KY742209	KY742140	KY742297
Didymella arachidicola	CBS 333.75 T	GU237833	GU237996	KT389598	GU237554
Didymella bellidis	CBS 714.85	GU237904	GU238046	KP330417	GU237586
Didymella brunneospora	CBS 115.58 T	KT389505	KT389723	KT389625	KT389802
Didymella chlamydospora	CGMCC 3.20072	MT229695	MT229672	MT239092	MT249263
Didymella coffeae-arabicae	CBS 123380 T	FJ426993	GU238005	KT389603	FJ427104
Didymella combreti	CBS 137982 T	KJ869134	KJ869191	MT018139	MT005626
Didymella curtisii	CBS 251.92	FJ427038	GU238013	MT018131	FJ427148
Didymella dactylidis	CBS 124513 T	GU237766	GU238061	MT018173	GU237599
Didymella degraaffiae	YMF1.05212	MH257408	MH257493	MH311843	OQ737922
Didymella degraaffiae	CBS 144956 T	MN823444	MN823295	MN824470	MN824618
Didymella dimorpha	CBS 346.82 T	GU237835	GU238068	MT018158	GU237606
Didymella erhaiensis	**YMF1.05023**	**MH257369**	**MH257457**	**MH311809**	**MH422997**
Didymella erhaiensis	**YMF1.05024**	**MH257366**	**MH257454**	**MH311806**	**MH422994**

Didymella erhaiensis	**YMF1.05084**	**MH257368**	**MH257456**	**MH311808**	**MH422996**
Didymella erhaiensis	**YMF1.05021**	**MH257367**	**MH257455**	**MH311807**	**MH422995**
Didymella eucalyptica	CBS 377.91	GU237846	GU238007	KT389605	GU237562
Didymella exigua	CBS 183.55 T	GU237794	EU754155	EU874850	GU237525
Didymella gardeniae	CBS 626.68 I T	FJ427003	GQ387595	KT389606	FJ427114
Didymella gei	YMF1.05051	MH257403	MH257488	-	OQ737923
Didymella gei	CGMCC 3.20068	MT229698	MT229675	MT239095	MT249266
Didymella glomerata	DF34	MH257370	-	MH311810	-
Didymella glomerata	JH24	MH257371	-	MH311811	MH422998
Didymella glomerata	CBS 528.66	FJ427013	EU754184	GU371781	FJ427124
Didymella gongkasis	**YMF1.05095**	**MH257372**	**MH257458**	**MH311812**	**MH422999**
Didymella gongkasis	**YMF1.05029**	**MH257373**	**MH257459**	**MH311813**	**MH423000**
Didymella guttulata	CBS 127976 T	MN973524	MN943730	MT018138	MT005625
Didymella heteroderae	CBS 109.92 T	FJ426983	GU238002	KT389601	FJ427098
Didymella hippuris	**YMF1.05089**	**MH257388**	**MH257473**	**MH311827**	**MH423015**
Didymella hippuris	**YMF1.05204**	**MH257397**	**MH257482**	**MH311835**	**-**
Didymella hippuris	**YMF1.05037**	**MH257381**	**MH257467**	**MH311821**	**-**
Didymella hippuris	**YMF1.05210**	**MH257374**	**MH257460**	**MH311814**	**-**
Didymella ilicicola	CGMCC 3.18355 T	KY742065	KY742219	KY742150	KY742307
Didymella indica	CBS 653.77 T	MN973534	MN943741	MT018159	MT005637
Didymella infuscatispora	CGMCC 3.18356 T	KY742067	KY742221	KY742152	KY742309
Didymella myriophyllana	**YMF1.05035**	**MH257399**	**MH257484**	**MH311837**	**MH423001**
Didymella keratinophila	CBS 143032 T	LT592901	LN907343	LT593039	LT592970
Didymella lethalis	CBS 103.25	GU237729	GU238010	KT389607	GU237564
Didymella ligulariae	CGMCC 3.20070	MT229699	MT229676	MT239096	MT249267
Didymella longicolla	CBS 124514 T	GU237767	GU238095	MT018161	GU237622

Didymella macrophylla	CGMCC 3.18357 T	KY742070	KY742224	KY742154	KY742312
Didymella macrostoma	CBS 223.69	GU237801	GU238096	KT389608	GU237623
Didymella maydis	CBS 588.69 T	FJ427086	EU754192	GU371782	FJ427190
Didymella maydis	CBS 247.60	MN973269	-	-	MN983897
Didymella microchlamydospora	CBS 105.95 T	FJ427028	GU238104	KP330424	FJ427138
Didymella mitis	CBS 443.72 T	MN973523	MN943729	MT018137	MT005624
Didymella myriophyllana	**GZ58**	**MH257402**	**MH257487**	**-**	**MH400427**
Didymella myriophyllana	**YMF1.05100**	**MH257401**	**MH257486**	**MH311839**	**MH423003**
Didymella negriana	CBS 358.71	GU237838	GU238116	KT389610	GU237635
Didymella nigricans	CBS 444.81 T	GU237867	GU238000	MT018146	GU237558
Didymella ocimicola	CGMCC 3.18358 T	KY742078	KY742232	MT018181	KY742320
Didymella pedeiae	CBS 124517 T	GU237770	GU238127	KT389612	GU237642
Didymella pinodella	CBS 531.66	FJ427052	GU238017	KT389613	FJ427162
Didymella pinodes	CBS 525.77 ET	GU237883	GU238023	KT389614	GU237572
Didymella pomorum	CBS 539.66 T	FJ427056	GU238028	KT389618	FJ427166
Didymella pomorum	CBS 354.52	MH857081	MH868616	KT389616	KT389799
Didymella prolaticolla	CBS 126182 T	MN973533	MN943740	MT018157	MT005636
Didymella prosopidis	CBS 136414 T	KF777180	KF777232	MT018149	MT005631
Didymella protuberans	CBS 381.96N T	GU237853	GU238029	KT389620	GU237574
Didymella qilianensis	LC 13584	MT229700	MT229677	MT239097	MT249268
Didymella qilianensis	CGMCC 3.20071	MT229701	MT229678	MT239098	MT249269
Didymella rhei	CBS 109177	GU237743	GU238139	KP330428	GU237653
Didymella rhei	BRIP 5562	KY742083	KY742237	KY742163	KY742325
Didymella sancta	CBS 281.83 T	FJ427063	GU238030	KT389623	FJ427170
Didymella segeticola	CGMCC 3.17489 T	KP330443	KP330455	KP330414	KP330399
Didymella senecionicola	CBS 160.78	GU237787	GU238143	MT018177	GU237657

Didymella sinensis	YMF1.05207	MH257407	MH257492	OQ737927	MH423007
Didymella sinensis	YMF1.05008	MH257405	MH257490	MH311842	MH423005
Didymella sinensis	CGMCC 3.18348 T	KY742085	KY742239	MT018127	KY742327
Didymella sinensis	CGMCC 3.18113	KX829033	KX829041	MT018126	KX829057
Didymella subglobispora	CBS 364.91 T	MN973531	MN943737	MT018153	MT005634
Didymella subglomerata	CBS 110.92	FJ427080	GU238032	KT389626	FJ427186
Didymella subherbarum	CBS 250.92 T	GU237809	GU238145	MT018162	GU237659
Didymella subrosea	CBS 733.79 T	MN973540	MN943747	MT018174	MT005643
Didymella suiyangensis	CGMCC 3.18352 T	KY742089	KY742243	KY742168	KY742330
Didymella suiyangensis	LC 8144	KY742090	KY742244	KY742169	KY742332
Didymella uniseptata	CGMCC 3.20069	MT229702	MT229679	MT239099	MT249270
Didymella variabilis	CBS 254.79 T	MN973544	MN943751	MT018182	MT005647
Didysimulans italica	MFLUCC 15-0059	KY496750	KY496730	KY514408	-
Didysimulans mezzanensis	MFLUCC 15-0067	KY496753	KY496733	KY514411	-
Dimorphoma isotiana	**YMF1.05048**	**MH257422**	**MH257507**	**MH311852**	**MH423018**
Dimorphoma saxea	CBS 419.92	GU237860	GU238141	KP330429	MT005727
Dimorphoma saxea	CBS 298.89	GU237824	GU238140	MT018299	GU237654
Ectodidymella nigrificans	CBS 100190	GU237708	GU237967	MT018078	GU237530
Ectodidymella nigrificans	PD 84/512	GU237919	GU237966	MT018077	GU237529
Ectophoma insulana	CBS 252.92	MN973481	MN943685	MT018070	MT005581
Ectophoma insulana	CBS 140548	MN973482	MN943686	MT018071	MT005582
Ectophoma multirostrata	YMF1.05209	MH257416	MH257501	OQ737928	MH423012
Ectophoma multirostrata	CBS 274.60	FJ427031	GU238111	LT623265	FJ427141
Ectophoma multirostrata	CBS 380.67	FJ427032	MN943683	MT018068	FJ427142
Ectophoma myriophyllanus	**YMF1.05050**	**MH257417**	**MH257502**	**MH311848**	**MH423013**
Ectophoma myriophyllanus	**YMF1.05208**	**MH257418**	**MH257503**	**MH311849**	**MH423014**

Ectophoma pomi	CBS 267.92	GU237814	GU238128	LT623263	GU237643
Ectophoma pomi	CBS 121.93	MN972933	MN973320	MN983570	MN983948
Epicoccum camelliae	CGMCC 3.18343	KY742091	KY742245	KY742170	KY742333
Epicoccum catenisporum	CBS 181.80	FJ427069	-	-	FJ427175
Epicoccum cedri	MFLU:16-1358	KY711170	KY711172	-	KY711168
Epicoccum dendrobii	CGMCC 3.18359	KY742093	KY742247	MT018084	KY742335
Epicoccum huancayense	YMF1.05047	MH257409	MH257494	MH311844	MH423008
Epicoccum huancayense	CBS 105.80	MH861244	MH873016	KT389630	GU237615
Epicoccum huancayense	CBS 390.93	GU237857	GU238085	MT018111	GU237616
Epicoccum latusicollum	KS52	MH257410	MH257495	MH311845	MH423009
Epicoccum latusicollum	CGMCC 3.18346*	KY742101	KY742255	KY742174	KY742343
Epicoccum latusicollum	LC 4859	KY742102	KY742256	KY742175	KY742344
Epicoccum longiostiolatum	CBS 902.96	MN973507	MN943713	MT018110	MT005607
Epicoccum mackenziei	MFLUCC 16-0335	KX698039	KX698028	KX698035	KX698032
Epicoccum mezzettii	CBS 173.38	MN973496	MN943701	MT018095	MT005596
Epicoccum nigrum	CBS 173.73	FJ426996	GU237975	KT389632	FJ427107
Epicoccum oryzae	CBS 174.34	MN973500	MN943705	MT018099	MT005600
Epicoccum ovisporum	CBS 180.80	FJ427068	LT623212	LT623252	FJ427174
Epicoccum plurivorum	KS55	MH257411	MH257496	OQ737929	MH423010
Epicoccum plurivorum	CBS 558.81	GU237888	GU238132	KT389634	GU237647
Epicoccum plurivorum	CBS 284.93	GU237822	GU238131	MN983591	GU237646
Epicoccum poaceicola	MFLUCC 15-0448	KX965727	KX954396	KX898365	KY197980
Epicoccum poae	LC 8161	KY742114	KY742268	KY742183	KY742356
Epicoccum poae	CGMCC 3.18363	KY742113	KY742267	KY742182	KY742355
Epicoccum polychromum	CBS 141502	MN973506	MN943712	MT018109	MT005606
Epicoccum proteae	CBS 114179	JQ044433	JQ044452	LT623251	LT623230

Epicoccum proteae	CBS 113875	MN973508	MN943714	MT018112	MT005608
Epicoccum pruni	MFLUCC 17-1059	NR 165862	NG 069437	-	-
Epicoccum purpurascens	CBS 124435	MN973486	MN943691	MT018081	MT005586
Epicoccum purpurascens	CBS 128906	MN973488	MN943693	MT018083	MT005588
Epicoccum sorghi	CBS 563.88	MN973504	MN943709	MT018106	MT005604
Epicoccum sorghinum	CBS 179.80	FJ427067	GU237978	KT389635	FJ427173
Epicoccum thailandicum	QC12	OQ421503	OQ421505	OQ737930	OQ737924
Epicoccum thailandicum	MFLUCC 16-0892	KY703619	KY703620	-	-
Epicoccum thailandicum	UTHSC: DI16-257	LT592927	LN907400	LT593065	LT592996
Epicoccum tobaicum	CBS 384.36	MN973493	MN943698	MT018092	MT005593
Epicoccum tritici	MFLUCC 16-0276	KX926426	KX954391	-	KY197979
Epicoccum variabile	CBS 119733	MN973501	MN943706	MT018103	MT005601
Epicoccum viticis	CGMCC 3.18344	KY742118	KY742272	KY742186	KY742360
Heterophoma nobilis	CBS 507.91	GU237877	GU238065	KT389638	GU237603
Heterophoma poolensis	CBS 116.93	GU237755	GU238134	MT018055	GU237649
Heterophoma sylvatica	CBS 874.97	GU237907	GU238148	MT018052	GU237662
Heterophoma verbascicola	CGMCC 3.18364	KY742119	KY742273	KY742187	KY742361
Heterophoma verbasci-densiflori	CBS 127.93	GU237774	GU238120	MT018051	GU237639
Juxtiphoma eupyrena	CBS 374.91	FJ426999	GU238072	LT623268	FJ427110
Juxtiphoma kolkmaniarum	CBS 146005	MN823568	MN823419	MN824593	MN824742
Juxtiphoma kolkmaniarum	CBS 527.66	FJ427000	GU238073	LT623269	FJ427111
Leptosphaerulina americana	CBS 213.55	MH857452	MH868995	KT389641	GU237539
Leptosphaerulina arachidicola	CBS 275.59	MH857863	MH869401	MT018278	GU237543
Leptosphaerulina australis	CBS 317.83	MH861604	MH873322	GU371790	GU237540
Leptosphaerulina australis	CBS 298.54	MH857343	MH868885	MN983618	MN983995
Leptosphaerulina briosiana	CBS 533.66	EU167575	MN943804	MT018266	MT005704

Leptosphaerulina chartarum	CBS 329.86	KJ796400	MH873651	MT018277	-
Leptosphaerulina gaeumannii	CBS 311.51	MN973601	MN943810	MT018274	MT005711
Leptosphaerulina gaeumannii	CBS 939.69	MH859488	MH871269	MT018273	GU237541
Leptosphaerulina gaeumannii	LC13568	MW090244	MW090671	MW092121	MW092140
Leptosphaerulina kasensis	**YMF1.05041**	**MH257414**	**MH257499**	**OQ737921**	**OQ737925**
Leptosphaerulina longiflori	MFLUCC19-0148	MK503800	MK503811	MK503805	-
Leptosphaerulina longiflori	FU310115	MK503801	MK503812	MK503806	-
Leptosphaerulina macrospora	CGMCC3.19693	MW090232	MW090672	MW092122	MW092141
Leptosphaerulina obtusispora	CBS 569.94	MN973602	MN943811	MT018275	MT005712
Leptosphaerulina saccharicola	YMF1.05076	MH257413	MH257498	OQ737931	OQ737926
Leptosphaerulina saccharicola	LC13561	MW090236	MW090664	MW092114	MW092133
Leptosphaerulina saccharicola	LC13565	MW090237	MW090668	MW092118	MW092137
Leptosphaerulina shangrilensis	**YMF1.05053**	**MH257415**	**MH257500**	**MH311847**	**MH423011**
Leptosphaerulina sisyrinchiicola	CBS 121688	MN973605	MN943814	MT018279	MT005715
Leptosphaerulina trifolii	CBS 235.58	MH857767	MH869300	MT018271	GU237542
Longididymella clematidis	CBS 123705	FJ515593	FJ515634	MT018076	FJ515611
Longididymella vitalbae	CBS 123706	MN973483	MN943687	MT018072	MT005583
Longididymella vitalbae	CBS 123707	FJ515595	FJ515636	MT018075	FJ515613
Macroventuria angustispora	CBS 502.72	GU237873	GU237985	MT018193	GU237545
Macroventuria anomochaeta	CBS 525.71	GU237881	GU237984	GU456346	GU237544
Macroventuria terrestris	CBS 127771	MN973550	MN943757	MT018194	MT005653
Macroventuria wentii	CBS 526.71	GU237884	GU237986	KT389642	GU237546
Microsphaeropsis fusca	CBS 116670	MN973573	MN943779	MT018220	MT005676
Microsphaeropsis olivacea	CBS 233.77	GU237803	GU237988	MT018217	GU237549
Microsphaeropsis viridis	CBS 432.71	GU237863	GU237987	MT018209	GU237548
Neoascochyta europaea	CBS 820.84	KT389511	KT389729	KT389646	KT389809

Neoascochyta exitialis	CBS 812.84	KT389517	KT389735	-	KT389815
Neoascochyta graminicola	CBS 102789	KT389518	KT389736	KT389649	KT389816
Neoascochyta paspali	CBS 560.81	FJ427048	GU238124	KP330426	FJ427158
Neoascochyta soli	CGMCC 3.18365	KY742121	KY742275	MT018306	KY742363
Neodidymelliopsis cannabis	CBS 121.75	GU237761	GU237972	MT018288	GU237535
Neodidymelliopsis moricola	MFLUCC 17-1063	KY684939	KY684941	KY684943	KY684937
Neodidymelliopsis negundinis	MFLUCC 18-0083	MG564165	MG564163	MG564166	MG564164
Neodidymelliopsis tiliae	CBS 519.95	MN973610	MN943819	MT018287	MT005721
Neomicrosphaeropsis elaeagni	MFLU 16-2389	MH069666	MH069672	MH069684	MH069691
Neomicrosphaeropsis italica	MFLUCC 15-0485	KU900318	KU729854	KU674820	-
Neomicrosphaeropsis tamaricicola	MFLUCC 14-0443	KU900322	KU729851	-	-
Nothophoma anigozanthi	CBS 381.91	GU237852	GU238039	KT389655	GU237580
Nothophoma arachidis-hypogaeae	CBS 125.93	GU237771	GU238043	KT389656	GU237583
Nothophoma brennandiae	CBS 145912	MN823579	MN823430	MN824604	MN824753
Nothophoma infossa	CBS 123395	FJ427025	GU238089	KT389659	FJ427135
Nothophoma pruni	MFLUCC 18-1600	MH827007	MH827028	-	-
Nothophoma quercina	CBS 633.92	GU237900	EU754127	KT389657	GU237609
Nothophoma variabilis	CBS 142457	LT592939	LN907428	LT593078	LT593008
Paraboeremia adianticola	CBS 187.83	GU237796	GU238035	KP330401	GU237576
Paraboeremia camelliae	CGMCC 3.18106	KX829034	KX829042	KX829050	KX829058
Paraboeremia litseae	CGMCC 3.18109	KX829029	KX829037	KX829045	KX829053
Paraboeremia putaminum	CBS 130.69	GU237777	GU238138	MT018186	GU237652
Paraboeremia putaminum	CBS 372.91	GU237843	GU238137	MT018188	GU237651
Paraboeremia rekkeri	CBS 144955	MN823511	MN823362	MN824537	MN824685
Paraboeremia selaginellae	CBS 122.93	GU237762	GU238142	MT018189	GU237656
Paraboeremia truiniorum	CBS 144952	MN823495	MN823346	MN824521	MN824669

Paramicrosphaeropsis ellipsoidea	CBS 197.97	MN973574	MN943780	MT018224	MT005680
Paramicrosphaeropsis ellipsoidea	CBS 194.97	MN973575	MN943781	MT018225	MT005681
Phoma herbarum	CBS 274.37	KT389537	KT389754	KT389662	KT389835
Phoma herbarum	CBS 127589	KT389539	KT389757	KT389664	KT389838
Phoma herbarum	CBS 615.75	FJ427022	EU754186	KP330420	FJ427133
Phomatodes aubrietiae	CBS 627.97	GU237895	GU238045	KT389665	GU237585
Phomatodes nebulosa	CBS 503.75	GU237875	GU238115	MN983673	GU237634
Phomatodes pilosa	CBS 628.68	MN973591	MN943797	MT018259	MT005697
Pleiochaeta setosa	CBS 496.63	EU167563	EU167563	MT018318	MT005742
Pleiochaeta setosa	CBS 118.25	KY929373	KY929376	MT018319	MT005743
Pseudoascochyta novae-zelandiae	CBS 141689	LT592892	LT592893	LT592895	LT592894
Pseudoascochyta pratensis	CBS 141688	LT223130	LT223131	LT223133	LT223132
Pseudopeyronellaea eucalypti	CPC 27678	KY979755	KY979810	KY979848	KY979921
Pseudopeyronellaea eucalypti	CPC 27682	KY979756	KY979811	KY979849	KY979922
Remotididymella anthropophila	CBS 142462	LT592936	LN907421	LT593075	LT593005
Remotididymella anthropophila	JZB380042	MN648210	MN640405	-	MN537431
Remotididymella bauhiniae	FMR 13770	MK347737	MK347954	MK434914	MK412884
Remotididymella brunnea	CBS 993.95	MN973476	MN943679	MT018064	MT005576
Remotididymella capsici	CBS 679.77	MN973478	MN943681	MT018066	MT005578
Remotididymella destructiva	CBS 378.73	GU237849	GU238063	LT623258	GU237601
Remotididymella hydrilanus	**YMF1.05022**	**MH257412**	**MH257497**	**MH311846**	**OQ737932**
Remotididymella humicola	CBS 120117	MN973477	MN943680	MT018065	MT005577
Stagonosporopsis actaeae	CBS 106.96*	GU237734	GU238166	KT389672	GU237671
Stagonosporopsis ailanthicola	MFLUCC 16-1439*	KY100872	KY100874	KY100876	KY100878
Stagonosporopsis andigena	CBS 101.80 (R)	GU237714	GU238169	-	GU237674
Stagonosporopsis andigena	CBS 269.80	GU237817	GU238170	MT018026	GU237675

Stagonosporopsis artemisiicola	CBS 102636 (R)	GU237728	GU238171	KT389674	GU237676
Stagonosporopsis astragali	CBS 178.25 (R)	GU237792	GU238172	MT018030	GU237677
Stagonosporopsis bomiensis	CGMCC 3.18366*	KY742123	KY742277	KY742189	KY742365
Stagonosporopsis bungeiana	**YMF1.05092**	**MH257419**	**MH257504**	**MH311850**	**MH422976**
Stagonosporopsis caricae	CBS 248.90	GU237807	GU238175	MT018023	GU237680
Stagonosporopsis caricae	CBS 282.76	GU237821	GU238177	MT018022	GU237682
Stagonosporopsis chrysanthemi	CBS 137.96	GU237783	GU238191	MT018011	GU237696
Stagonosporopsis chrysanthemi	CBS 500.63	GU237871	GU238190	MT018012	GU237695
Stagonosporopsis citrulli	CBS 214.65	MN973454	MN943656	MT018020	MT005553
Stagonosporopsis crystalliniformis	CBS 713.85*	GU237903	GU238178	KT389675	GU237683
Stagonosporopsis cucumeris	CBS 386.65*	MN973455	MN943657	MT018021	MT005554
Stagonosporopsis cucurbitacearum	CBS 133.96	GU237780	GU238181	KT389676	GU237686
Stagonosporopsis cucurbitacearum	CBS 233.52	MN973456	MN943658	MT018024	MT005555
Stagonosporopsis dennisii	CBS 631.68*	GU237899	GU238182	KT389677	GU237687
Stagonosporopsis dorenboschii	CBS 426.90*	GU237862	GU238185	KT389678	GU237690
Stagonosporopsis helianthi	CBS 200.87*	KT389545	KT389761	KT389683	KT389848
Stagonosporopsis heliopsidis	CBS 109182 (R)	GU237747	GU238186	KT389679	GU237691
Stagonosporopsis hortensis	CBS 104.42 (R)	GU237730	GU238198	KT389680	GU237703
Stagonosporopsis inoxydabilis	CBS 425.90*	GU237861	GU238188	KT389682	GU237693
Stagonosporopsis inoxydabilis	MF-9.239	MH651539	MH651580	-	MH725216
Stagonosporopsis lupini	CBS 101494*	GU237724	GU238194	KT389685	GU237699
Stagonosporopsis malaiana	**YMF1.05087**	**MH257421**	**MH257506**	**MH311851**	**MH423017**
Stagonosporopsis oculo-hominis	CBS 634.92*	GU237901	GU238196	KT389686	GU237701
Stagonosporopsis papillata	CGMCC 3.18367*	KY742125	KY742279	KY742191	KY742367
Stagonosporopsis pini	MFLUCC 18-1549	MK347800	MK348019	MK434860	MK412886
Stagonosporopsis rudbeckiae	CBS 109180 (R)	GU237745	GU238197	MT018015	GU237702

Stagonosporopsis sambucella	CBS 130003	MN973459	MN943661	MT018029	MT005558
Stagonosporopsis stuijvenbergii	CBS 144953*	MN823449	MN823300	MN824475	MN824623
Stagonosporopsis stuijvenbergii	JW 33021	MN823450	MN823301	MN824476	MN824624
Stagonosporopsis tanaceti	YMF1.05015	MH257420	MH257505	-	MH423016
Stagonosporopsis tanaceti	CBS 131484*	NR 111724	JQ897461	MT018013	JQ897496
Stagonosporopsis tanaceti	CBS 131485	MN973452	MN943654	MT018014	MT005551
Stagonosporopsis valerianellae	CBS 329.67*	GU237832	GU238201	MT018034	GU237706
Stagonosporopsis weymaniae	CBS 144959	MN823453	MN823304	MN824479	MN824627
Vacuiphoma bulgarica	CBS 357.84	GU237837	GU238050	LT623256	GU237589
Vacuiphoma laurina	CBS 119636	MN973551	MN943758	MT018195	MT005654
Vacuiphoma oculihominis	UTHSC: DI16-308	LT592954	LN907451	LT593093	LT593023
Vandijckomycella joseae	CBS 144948	MN823589	MN823440	MN824614	MN824763
Vandijckomycella joseae	CBS 143011	MN823590	MN823441	MN824615	MN824764
Vandijckomycella snoekiae	CBS 144954	MN823591	MN823442	MN824616	MN824765
Xenodidymella applanata	CBS 205.63	GU237798	MH869871	KP330402	GU237556
Xenodidymella applanata	CBS 195.36 T	KT389548	KT389764	MT018280	KT389852
Xenodidymella asphodeli	CBS 375.62	KT389549	MH869779	KT389689	MT005716
Xenodidymella asphodeli	CBS 499.72	KT389550	KT389766	MT018282	KT389853
Xenodidymella camporesii	MFLUCC 17–2309	NR169976	MN244168	-	MN871955
Xenodidymella catariae	CBS 102635	GU237727	GU237962	KP330404	GU237524
Xenodidymella glycyrrhizicola	CBS 141234	MN973607	KX342932	MT018284	MT005718
Xenodidymella glycyrrhizicola	CBS 684.97	MN973606	MN943815	MT018283	MT005717
Xenodidymella humicola	CBS 220.85	GU237800	GU238086	KP330422	GU237617
Xenodidymella menthae	SCUA-Ahm-W4	OK257018	OK257026	OK247739	OK247745
Xenodidymella menthae	SCUA-Ahm-W4-2	OK257019	OK257027	OK247740	OK247746
Xenodidymella weymaniae	CBS 144960	MN823588	MN823439	MN824613	MN824762

Coniothyrium palmarum	CBS 400.71	AY720708	EU754153	KT389592	KT389792
